# Epigenetics of Pluripotent Cells 

**Published:** 2012

**Authors:** S.P. Medvedev, E.A. Pokushalov, S.M. Zakian

**Affiliations:** Institute of Cytology and Genetics, Siberian Branch, Russian Academy of Sciences, Prospekt Lavrentyeva, 10, Novosibirsk, Russia, 630090; Meshalkin Novosibirsk State Research Institute of Circulation Pathology, Rechkunovskaja Str., 15, Novosibirsk, Russia, 630055; Institute of Chemical Biology and Fundamental Medicine, Siberian Branch, Russian Academy of Sciences, Prospekt Lavrentyeva, 8, Novosibirsk, Russia, 630090

**Keywords:** embryonic stem cells, induced pluripotent stem cells, pluripotency, covalent histone modifications, DNA methylation

## Abstract

Pluripotency is maintained by a complex system that includes the genetic and epigenetic levels. Recent studies have shown that the genetic level (transcription factors, signal pathways, and microRNAs) closely interacts with the enzymes and other specific proteins that participate in the formation of the chromatin structure. The interaction between the two systems results in the unique chromatin state observed in pluripotent cells. In this review, the epigenetic features of embryonic stem cells and induced pluripotent stem cells are considered. Special attention is paid to the interplay of the transcription factors OCT4, SOX2, and NANOG with the Polycomb group proteins and other molecules involved in the regulation of the chromatin structure. The participation of the transcription factors of the pluripotency system in the inactivation of the X chromosome is discussed. In addition, the epigenetic events taking place during reprogramming of somatic cells to the pluripotent state and the problem of “epigenetic memory” are considered.

## INTRODUCTION 

Pluripotency is the property of cells to differentiate into the derivatives of all three primary germ layers – ectoderm, endoderm, and mesoderm - as well as to form precursor cells of functional gametes during embryonic development. Inner cell mass (ICM) cells and epiblast cells from pre-implantation mammalian embryos are pluripotent [1]. The adult organism is formed from pluripotent cells during ontogenesis. However, these cells cannot give rise to extraembryonic organs and tissues. 

Embryonic stem cells (ESCs) are obtained from the inner cell mass of pre-implantation embryos [2–4]. Under optimal cultivation conditions, ESCs can retain a number of properties intrinsic to the inner cell mass and embryonic epiblast cells (including pluripotency) for a long period [2–4]. The pluripotency makes ESCs promising for use in fundamental and applied research. ESCs are used as model systems to study the processes occurring during early embryogenesis in mammals and to *in vitro* simulate various diseases. Furthermore, pluripotent cells are a promising source of material for substitutive cellular therapy [5–7]. 

After the first mouse and human ESC lines were obtained, research into the molecular genetic basis involved in maintenance of the undifferentiated pluripotent state of ESCs started. It is known today that the pluripotent state of the cells of pre-implantation embryos and ESCs is maintained via a complex system of cell surface proteins, their molecular signal pathways, and the transcription factors that initiate the transcription of the target genes. The subsystem of the so-called “external regulators of pluripotency” includes several signaling pathways, among which the cascades triggered by the proteins LIF, BMP4, TGFβ, activin A, NODAL, and bFGF (FGF2) are the major ones [1]. 

ESC pluripotency is also controlled by the subsystem of “internal regulators of pluripotency” – transcription factors functioning in cell nuclei. The factors OCT4, NANOG, and SOX2 are among the key regulators in this subsystem [8, 9]. 

In 2006, the data on reprogramming of mouse somatic cells into the pluripotent state were published in *Cell* by a group of Japanese researchers [10]. This was one of the most outstanding discoveries of the past decade in the field of cell biology. Cells obtained by the reprogramming of somatic cells were called induced pluripotent stem cells (iPSCs) [10]. 

The development of the technology for obtaining animal and human induced pluripotent stem cells has opened up a broad range of possibilities for studying the dynamics of the epigenetic events occurring upon reprogramming and the features of the epigenomes of pluripotent cells. A large number of well-reproducible methods for obtaining iPSCs from a broad range of somatic cells are known today. Most researchers use a certain gene combination for reprogramming; many of these genes encode transcription factors (e.g., *Oct4, Sox2, Klf4, c-Myc, Nanog,* and *Lin28 * genes) [10–13]. Furthermore, it has been demonstrated that mouse and human iPSCs can be obtained using miRNA [14, 15]. iPSCs have been successfully derived from various types of somatic cells. iPSCs were first obtained from fibroblasts of different origins, and subsequently from keratinocytes, melanocytes, blood cells, neural stem cells, pancreatic β-cells, B lymphocytes, and other cells [16–22]. Thus, it can be concluded that iPSCs can be derived from cells originating from all three primary germ layers (ectoderm, mesoderm, and endoderm), although efficiency in and the dynamics of the derivation of stable iPSC lines considerably depends on the method used and type of somatic cells [14, 23]. iPSCs obtained as a result of direct reprogramming have a number of common properties, which makes them such promising models for studies in the field of the biology of pluripotent cells and enables to use them to simulate human diseases and in regenerative medicine [6, 7]. In terms of their properties, induced pluripotent stem cells are very close to embryonic stem cells, which are derived from mouse and human pre-implantation embryos. Both cell types possess a similar morphology, sensitivity to growth factors and signaling molecules, and patterns of gene expression and differentiation [24]. In particular, during *in vitro* differentiation, iPSCs can form embryoid bodies consisting of the derivatives of all three germ layers. Furthermore, human iPSCs can form teratomes, whereas mouse iPSCs give rise to chimeras and are even capable of forming an entire organism when injected into tetraploid blastocysts [25–27]. It is obvious that all these properties typical of pluripotent cells are determined by the special state of epigenome, which is “inherited” by ESCs from the inner cell mass cells or is formed during reprogramming in the case of iPSCs. 

Recent studies have demonstrated that transcription factors, signaling pathways, and miRNA closely interact with the system of enzymes and other specific proteins that participate in the formation of the chromatin structure. The unique state of chromatin in pluripotent cells is formed by this interplay. 

The features of the epigenomes of embryonic stem cells and induced pluripotent stem cells are considered in this review. Special attention is focused on the interaction of the transcription factors OCT4, SOX2 and NANOG with Polycomb group proteins and the other molecules that participate in the regulation of the chromatin structure. The participation of the transcription factors of the system of pluripotency maintenance during the process of X chromosome inactivation is discussed. Moreover, the epigenetic events occurring upon reprogramming of somatic cells to a pluripotent state and the problems associated with the “epigenetic memory” are considered. 

## BIVALENT CHROMATIN DOMAINS IN PLURIPOTENT CELLS 

Chromatin regions simultaneously enriched in marks of active and inactive chromatin (H3К4me3 and H3К27me3) are known as bivalent domains [28]. These domains were found in mouse and human ESCs [28–31]. The genes whose transcription start sites are associated with the bivalent domains are characterized by a low transcription level regardless of the presence of the active chromatin mark, which attests to the fact that H3К27me3 prevails over H3К4me3. A high level of the histone H2A variant H2AZ was detected in bivalent domains [32]. Most bivalent domains are connected with the transcription start sites of the genes associated with development, e.g., transcription factors of the families HOX, SOX, FOX, PAX, IRX, and POU [28]. During the differentiation, most bivalent domains become monovalent and contain either H3К27me3 or H3К4me3 depending on the type of differentiated derivatives [28, 33]. However, some domains remain in their bivalent state and are present in the epigenomes of precursor cells [33, 34]. In general, the existence of bivalent domains and preservation of active chromatin marks in the promoter regions of the genes involved in maintaining an undifferentiated state allows one to quickly switch between programs of gene transcription upon differentiation to certain derivatives. 

## Interplay OF transcription factors of the system of pluripotency maintenance with polycomb group proteins and chromatin remodeling factors 

The existence of the so-called open chromatin in ESCs and simultaneous reliable repression of the differentiation genes are provided by the interaction system both at the protein – DNA and protein – protein levels. The investigation into the proteome of pluripotent cells and, in particular, the proteins forming the main system of pluripotency maintenance (OCT4, NANOG, SOX2) has shown that proteins not only interact with one another, thus regulating the transcription of a number of genes, but that they also form a complex interaction network with the other transcription factors and proteins that participate in chromatin modification and remodeling. The proteins involved in pluripotency maintenance interact with the components of protein complexes, such as PRC1 and 2, BAF, NuRD, etc. [35–38]. 

## POLYCOMB GROUP PROTEINS. COMPLEXES PRC1 AND 2 

Polycomb group proteins are an evolutionary-conserved family of regulators of the chromatin structure. The role of these proteins is to achieve and maintain the transcriptional silencing of homeotic genes [39–41]. 

Two complexes belonging to the Polycomb family are known in mammals: PRC1 (Polycomb Repressive Complex 1) and PRC2, which play an essential role in embryonic development and in the maintenance of stem cell self-renewal and normal differentiation. 

The mammalian PRC1complex consists of several subunits; homologues of these were found in Drosophila: CBX1, 2 and 3; MEL18, Bmi1, RING1A (RING1), RING1B (RNF2) and PHC1, 2 and 3. It is considered that the role of PRC1 is to maintain the genes in their repressed state, which is originally achieved by the PRC2 complex. This function is activated through the activity of the subunits RING1A and 1B, which belong to the E3 ligase family and perform monoubiquitination of H2A histone at K119 (H2AK119Ub1) [42–44]. Mice having mutations in the PRC1 subunit genes (except for RING1B) remain alive, which may attest either to the existence of alternative mechanisms or to redundancy of the PRC1 function for the normal regulation of embryonic development [45]. However, it has been ascertained that the components of the PRC1 complex (e.g., BMI1) are required to ensure the functioning of several types of regional stem cells (hematopoietic, neural, lung and intestinal stem cells) [46–49]. It is of interest that the function of Bmi1 and PRC1 in regional stem cells is presumed to be confined to control over the system regulating the level of reactive oxygen in mitochondria [50]. Furthermore, the absence of RING1A and 1B causes spontaneous differentiation of mouse ESCs and activates the genes associated with differentiation and development. Interestingly, the promoters of a large number of genes repressed by PRC1 are bound to the OCT4 transcription factor, which also participates in the transcriptional repression of these genes. Binding of PRC1 to the target genes depends on OCT4, whereas binding of OCT4 is PRC1-independent [51]. Proteomic studies have demonstrated that RING1B (RNF2) physically interacts with the NANOG transcription factor in ESCs [37]. These facts indicate that there is a close relationship between the system of transcription factors that maintain pluripotency and the system of regulators of the chromatin structure (in particular, PRC1). 

**Fig. 1:**
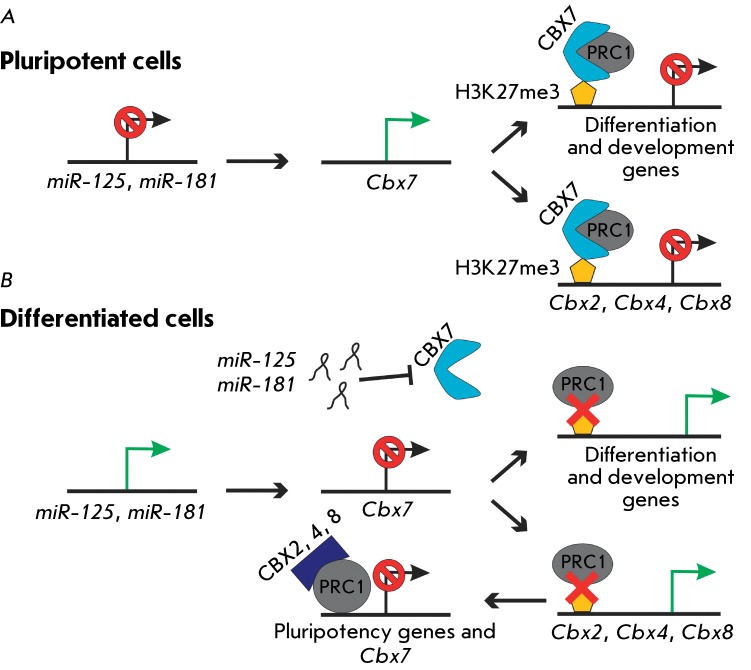
Model illustrating the role of CBX proteins in PRC1 regulation in pluripotent cells and during differentiation. (A) In pluripotent cells, the CBX7/PRC1 complex binds to the regulatory regions of the genes involved in development and differentiation and the genes encoding the CBX2, 4, and 8 proteins, which represses their transcription. This binding depends on H3K27me3 established by PRC2. (B) Expression of microRNAs *miR-125* and *miR-181* , which repress CBX7 expression, is activated during differentiation. The absence of CBX7/PRC1 results in activation of the differentiation genes, as well as *Cbx2* , *4* and *8* . PRC1, together with CBX2, CBX4, and CBX8, represses the transcription of the genes responsible for pluripotency maintenance and *Cbx7* [55]

A new function of CBX proteins, components of the PRC1 complex, in the regulation of the self-renewal and differentiation of mouse ESCs has been recently detected [52, 53] ( *Fig. 1* ). Five CBX proteins associated with PRC1 – Cbx2, Cbx4, Cbx6, Cbx7, and Cbx8 are known in mammals [54]. The methods of ChIP-Seq (chromatin immunoprecipitation followed by sequencing of enriched DNA) and co-immunoprecipitation were used to demonstrate that in undifferentiated mouse ESCs, 97% of the CBX7 binding sites contain the complexes PRC1 and PRC2; 86% of them are also marked by H3K27me3. Several sites are located within the development-associated genes (e.g., sites in the HOX gene cluster [52]). 

It has also been demonstrated using a quantitative proteomic analysis that only CBX7 co-localizes with H3K27me3 in undifferentiated mouse ESCs, whereas CBX2 and CBX8 interact with this histone modification in differentiated cells and fibroblasts [53]. Furthermore, it has been established via chromatin immunoprecipitation that CBX7 in a complex with PRC1 interacts with the *Cbx2, Cbx4* and *Cbx8 * gene promoters, suppressing their transcription in ESCs [52]. Contrariwise, Cbx2, Cbx4 and Cbx8, which can participate in *Cbx7 * expression, bind to PRC1 during the differentiation process [52, 53]. Suppression of *Cbx7 * expression in ESCs results in enhanced expression of the * Cbx2, Cbx4* , and *Cbx8* genes, disruption of the ESC morphology, and spontaneous differentiation. Ectopic increased *Cbx7 * expression suppresses differentiation and X chromosome inactivation in female cells and enhances their self-renewal [53]. In addition, *miR-125* and *miR-181* participate in the suppression of *Cbx7* transcription, which supports the fact that miRNAs play a significant role in the regulation of the Polycomb protein function [53]. Thus, the dynamic system of PRC1 and CBX proteins, which are mutually regulated, participates in the regulation of the self-renewal and differentiation of ESCs. The function of these complexes is regulated by PRC2 (H3K27me3); their combinations change depending on cell status ( *Fig. 1* ). 

The mammalian protein complex PRC2 contains the EED (embryonic ectoderm development), SUZ12 (suppressor of zeste 12), and EZH1 (enhancer of zeste 1) or EZH2 (enhancer of zeste 2) subunits. EZH2 is the protein with the SET domain, which is attributable to the proteins functioning as histone methyltransferases and performs di- and trimethylation of histone H3 at K27 (H3K27me2/3). As opposed to PRC1, gene mutations of PRC2 subunits cause significant disruptions in embryonic development and embryonic death [45, 56, 57]. Disrupted gastrulation (the pattern of the germinal streak is changed in the anteroposterior direction), hypertrophied extraembryonic mesoderm, and undeveloped embryonic mesoderm are observed in embryos with the mutant * Eed* gene and the absence of H3K27 methylation [57, 58]. However, *Eed* mutant blastocysts can be used to obtain ESCs possessing pluripotency but tending towards spontaneous differentiation [59]. A similar situation has also been observed for *Suz12 * mutants. Despite the fact that the death of * Suz 12 * mutant mouse embryos is observed, ESCs can be successfully obtained. Although ESCs obtained from the mutant embryos possess a high level of transcription of the differentiation genes, they do not form neurons during *in vitro* differentiation and slightly differentiate to endoderm derivatives when embryoid bodies are formed [56]. Deletion of the *Ezh2* gene results in no changes in the properties of ESCs obtained from mutant embryos; this fact can be attributed to the effect of the EZH1 subunit, which also has histone–methyltransferase activity and mediates the setting of the mark of inactive chromatin within the PRC2 target genes [60]. 

It has been recently found that the JARID2 protein from the family JUMONJI C (JMJ C) is one of the subunits of the PRC2 complex. JUMONJI proteins belong to histone demethylases; however, JARID2 lacks such activity. It has been shown that JARID2 is required to provide efficient binding of PRC1 and PRC2 to the promoters of the target genes; the binding pattern of PRC2 and JARID2 to the DNA of the target genes in the scale of the genome of mouse ESCs coincides to over 90% [61–66]. There is abundant controversial experimental data on the effect of *Jarid2* knockout or knockdown on the H3K27me3 level in promoters of the PRC2 target genes. A decrease in the H3K27me3 level has been observed in some studies [63, 65, 66], whereas both the absence of changes [61] and an increase in the H3K27me3 level [62] have also been reported. However, the differentiation process has been shown to be disrupted or decelerated in JARID2-deficient ESCs; i.e., JARID2 affects pluripotency in some way [62, 63, 66]. In addition, JARID2, jointly with MTF2 and esPRC2p48 proteins, is capable of enhancing efficiency in obtaining induced pluripotent stem cells from mouse embryonic fibroblasts via overexpression of the *Oct4* , *Sox2* , and *Klf4* genes. On the contrary, knockout of the genes encoding JARID2, MTF2, and esPRC2p48 considerably suppresses reprogramming [67]. 

**Fig. 2:**
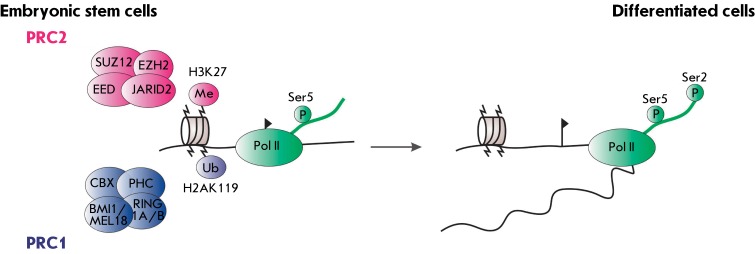
JARID2 is necessary to recruit the Ser5-phosphorilated form of RNA-polymerase II in the bivalent domains of mouse ESC epigenome. In pluripotent cells, the formation of “bivalent domains” requires RNA-polymerase II phosphorylated at Ser5 (green oval) in the promoter regions of the genes whose expression is repressed by PRC2. PRC2 subunits mediate H3K27 methylation; in turn, it recruits PRC1 responsible for H2AK119 monoubiquitylation [68]

There are several hypotheses on the molecular basis of the effect of JARID2 on cell pluripotency; none of those has been sufficiently supported through experimental data [68]. The major role of JARID2 is believed to be not in the modulation of the histone-methyltransferase activity of PRC2, but in recruiting a special initiating form of RNA polymerase II [66, 68] ( *Fig. 2* ). This form of RNA polymerase has a phosphorylated serine residue at position 5 (Ser5P-RNAP) (while in the elongating form, the serine residue at position 2 is also phosphorylated); the presence of this form is typical of bivalent epigenome domains, which are formed with the participation of PRC1 and PRC2 [69, 70]. The presence of this polymerase form within promoters of the genes participating in cell differentiation appears to be required for rapid and reliable switching between transcription programs when the differentiation process is initiated. 

Thus, it can be concluded that PRC2 plays an essential role in the regulation of mammalian development and ESC differentiation; however, this complex does not affect the process of obtaining ESCs and their self-renewal. Abundant experimental evidence of joint regulation of the target genes by PRC2 and transcription factors OCT4, SOX2, and NANOG, which are the central part of the system of gene transcription regulation in mouse and human ESCs, has been obtained. Whole-genome studies have demonstrated that OCT4, SOX2, NANOG, and the PRC2 subunits colocalize in the genes responsible for development, intracellular signal transfer, morphogenesis and organogenesis; hence, they can function jointly [8, 28, 71, 72]. 

## Trithorax (trxG) COmplex 

The proteins of the Trithorax complex are among the major regulators of the embryonic development of both invertebrates and vertebrates [73]. The role of Trithorax during their development is opposite to that of Polycomb proteins; they mediate H3K4me3, which is mostly associated with transcription activation. Unlike the PRC1 and PRC2 complexes, the role of Trithorax proteins in maintaining cell pluripotency remains poorly studied [74]. Identically to that in invertebrates, Trithorax in mammals is a multi-subunit complex containing histone methyltransferases Set/MLL. The main subunits of the complex, Wdr5, Ash2l and Rbbp5, are needed to activate the Set/MLL enzymes [75]. The ASH2L/RBBP5 heterodimer is known to directly contribute to the histone methyltransferase activity of the MLL1 complex [76]. Furthermore, experimental evidence of the fact that ASH2L is needed for normal embryogenesis and X chromosome inactivation in female mice has been obtained [77]. The Wdr5 subunit is also the major component of the mammalian Trithorax complex. Its function consists in “providing” H3K4 residues and in carrying out an efficient interaction between the entire Trithorax complex and H3K4, and thus in implementation of its histone-methyltransferase activity [75]. Furthermore, Wdr5 is known to recognize H3K4me2 and mediate the transition of H3K4 into a trimethylated state (H3K4me3) [78]. It has recently been established that WDR5 is required not only to ensure the normal development of vertebrates, but also plays a crucial role in maintaining ESC pluripotency and cell reprogramming to a pluripotent state [74]. The inhibition of WDR5 expression was ascertained to abruptly reduce the self-renewal of mouse ESCs. Proteomic studies have allowed to establish that WDR5 physically interacts with OCT4 in undifferentiated ESCs, so that the targets of these two proteins overlap to a significant extent. Thus, it has been demonstrated that the Trithorax complex, along with OCT4, SOX2 and NANOG, positively regulates gene transcription in mouse ESCs. Furthermore, it has been shown in experiments on the reprogramming of somatic cells that the Trithorax complex (WDR5) is required to provide efficient formation of iPSC clones [74]. 

## BAF COMPLEX 

Numerous studies have shown that ATP-dependent chromatin remodeling protein complexes play an essential role in the embryonic development of mammals in general and in maintaining cell pluripotency in particular [79–84]. Nearly 30 proteins possessing ATP-dependent chromatin-remodeling activity have been known in mammals. These proteins have been grouped into several families in accordance with the structure of the ATPase domain. In mammalian cells, chromatin-remodeling ATPases interact with each other and the other proteins and act within protein complexes consisting of several subunits. BAF, NuRD, ISWI can be mentioned as examples of such complexes. The BAF protein complex participates in the redistribution of nucleosomes and is present in all cell types. However, the subunit composition of this complex may vary for different cell types, and chromatin structure is controlled in a fashion specific to each cell type. ESCs contain the BAF complex (known as esBAF), which in turn consists of a specific combination of the subunits BRG, BAF155, and BAF60a but contains no subunits Brm, BAF170, or BAF60c [83, 84]. It has been experimentally demonstrated that the inactivation of most subunits of the BAF complex causes the death of mouse embryos at the early stages of development and also leads to cellular oncotransformation [79, 85–88]. Furthermore, embryonic death in case of loss of the subunits Brg, BAF47, and BAF155 has been caused by the disturbance of the formation of pluripotent cells. Careful screening of libraries of interfering RNAs has demonstrated that such subunits as Brg and BAF155 are required to maintain the morphology of ESC colonies and expression of *Nanog* [89, 90]. According to proteomic data, several subunits of chromatin-remodeling complexes physically interact with OCT4 and NANOG in ESCs [35–38, 83, 84]. 

The transcription factors OCT4 and NANOG can interact with chromatin remodeling complexes via specific proteins [37, 90]. Thus, it has been demonstrated that the chromosome scaffold protein TIF1b (Transcription Intermediary Factor-1b) is required for maintaining the activity of the *GFP* transgene in ESCs under the control of the *Oct4* promoter [92]. Interestingly, TIF1b used to be known as a protein that participates in transcriptional silencing and heterochromatin formation through recruitment of the heterochromatin protein HP1 and histone methyltransferase SETDB1 and NuRD. However, the phosphorylated form of TIF1b can interact with the ESC-specific form of the BAF complex, localized in euchromatin, and is capable of affecting the efficiency of the induced pluripotent stem cell derivation [91]. Furthermore, overexpression of ESC-specific components of this complex, Brg1 and BAF155, increases the efficiency of the reprogramming of somatic cells in the absence of *c-Myc* overexpression [93, 94]. 

It has recently been demonstrated that the esBAF complex is directly associated with the activity of the LIF-STAT3 signaling pathway, which is necessary for maintaining pluripotency of mouse ESCs [95, 96]. The transcription factor STAT3 is known to activate gene groups in various cell types containing specific BAF complexes; however, it is only in ESCs that it contributes to the regulation of the target genes required to maintain an undifferentiated status of ESCs. However, the mechanism of such a specific effect of STAT3 remained unclear for a long time. 

**Fig. 3:**
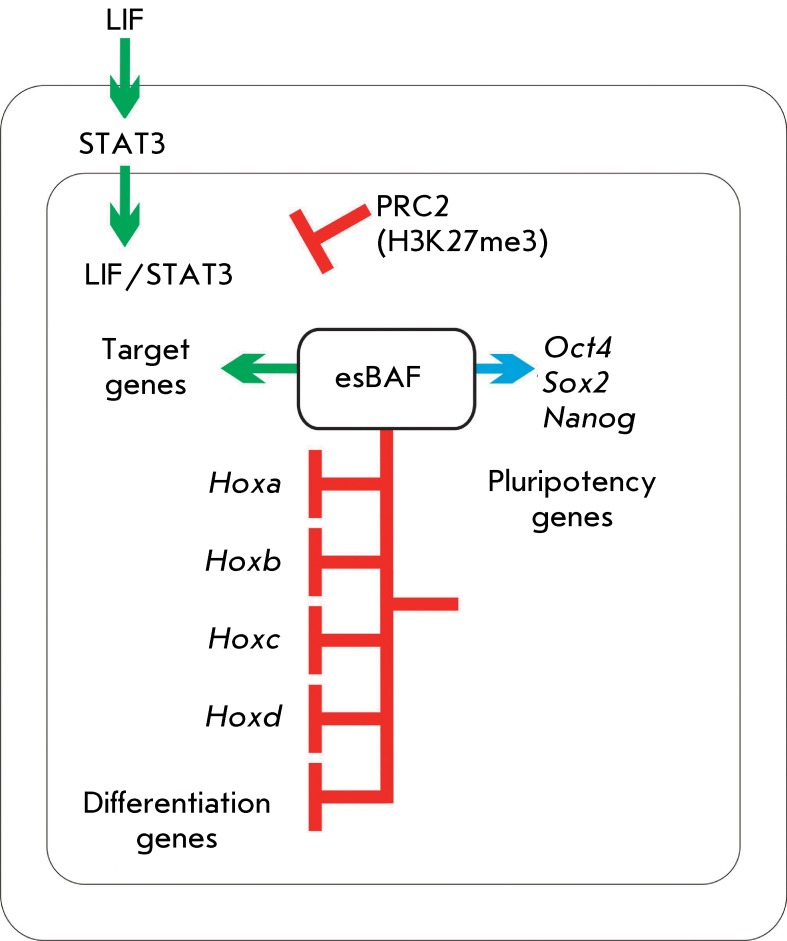
Cooperation of esBAF and PRC2 during pluripotency maintenance. esBAF and PRC2 can act both synergically and antagonistically. esBAF antagonizes PRC2 when regulating the target genes of the LIF-STAT3 signaling pathway, thus preparing the chromatin structure for activation by the phosphorylated STAT3 form (green arrow). Meanwhile, esBAF acts together with PRC2 to repress transcription of HOX genes (red lines with stoppers). However, expression of pluripotency genes can be activated or repressed by esBAF (blue arrow) [96]

L. Ho *et al.* [96] have ascertained that binding of STAT3 to the target sites in the genome of mouse ESCs depends on BRG1, the ATPase subunit of the ESC-specific esBAF complex. The effect of BRG1 within STAT3-binding sites forms the chromatin structure, which is required for gene activation by interleukin LIF. BRG1 deletion induces PRC2-mediated transcriptional silencing of a number of genes at the level of the entire genome via H3K27me3. STAT3 targeted genes undergo transcriptional silencing as well. Based on these facts, a conclusion has been drawn that the major role of Brg1 in mouse ESCs is enhancing the action of the LIF-STAT3 signaling pathway and counteracting the repression of this pathway by the Polycomb proteins (PRC2). It is an interesting fact that BRG1 can act jointly with Polycomb to enhance repression of the differentiation genes (e.g., HOX family genes). Thus, the esBAF complex can act both antagonistically and synergically with PRC2; however, both types of actions work towards the maintenance of pluripotency [96] ( *Fig. 3* ). 

## NuRD COMPLEX 

The mammalian protein complex NuRD (Nucleosome Remodeling Deacetylase), which exhibits ATP-dependent remodeling and histone deacetylase activity, consists of at least six subunits [97, 98]. NuRD contains histone acetylases HDAC1 and HDAC2, whose activity is dependent on the chromodomain-containing ATPase subunits Mi2a and Mi2b. In addition, the complex contains proteins binding methylated cytosine Mbd 1, 2 and 3 (methyl-CpG-binding proteins), proteins MTA1, 2 and 3 (Metastasis-associated proteins), WD40-containing proteins RbAP46 and RbAP48, as well as two proteins containing zinc finger domains (p66a and p66b). Several subunits of the NuRD complex have been demonstrated to be required to maintain the pluripotency and differentiation of ESCs. Embryonic stem cells with a deletion of the gene encoding Mbd3 retain their viability and expression of pluripotency markers; however, they cannot differentiate both *in vitro * and *in vivo* upon formation of chimeric animals [99]. Nevertheless, it has been demonstrated in one of the later studies that *Mbd3* knockout in mouse ESCs enhances the transcription level of such trophoblast markers as *Cdx2* , *Eomesodermin,* and *Hand1* and the acetylation level of histone H3 in the promoter regions of these genes. Furthermore, knockout cells grown in media for trophoblast stem cells have differentiated into trophoblast cells expressing CDX2 and Cadherin 3 [100]. It has been shown in *in vivo * experiments that Mbd3 is required for the development of epiblast from ICM cells after implantation. In Mbd3-deficient embryos, the pluripotency genes *Oct4* , *Nanog* and *Sox2, * as well as their target genes, are expressed at the normal level; however, their normal transcriptional silencing can be disturbed after the implantation. On the contrary, the cultured ICM of MDB3-deficient embryos cannot give rise to pluripotent ESC lines, although they form a significant number of endodermal derivatives [101]. 

Mouse ESCs contain a specific subfamily of NuRD complexes known as NODE (NANOG and OCT4 associated deacetylase). The NODE complex consists of histone deacetylases HDAC1 and HDAC2, as well as Mta1 and 2. However, this complex contains almost no MBD3 or RBBP7 subunits (they are detected in substoichiometric amounts). NODE is of interest because it physically interacts with the OCT4 and NANOG transcription factors in mouse ESCs [35]. NODE exhibits deacetylase activity, which is MBD3-independent. A knockout of the genes encoding the NODE subunits results in an enhancement of the expression of the genes responsible for differentiation; hence, it causes differentiation of ESCs into various cell derivatives. It has been demonstrated in experiments aimed at translation inhibition that, unlike MBD3 which is required to repress transcription of the genes maintaining the undifferentiated state, MTA1 participates in the inhibition of the differentiation genes, such as *Gata6 * and *FoxA2 * [35] *.* Thus, ESCs contain at least two subfamilies of NuRD complexes that act in opposite directions: 1) MBD3-containing complexes that regulate (inhibit) the transcription of the pluripotency genes ( *Oct4* , *Nanog* and *Sox2* ,etc.) and are required for ESC differentiation into various cellular derivatives and for cell differentiation during early embryonic development; 2) HDAC1-, HDAC2-, and Mta1-containing complexes interacting with OCT4 and NANOG and participating in the transcription activation of the genes responsible for the maintenance of an undifferentiated state. 

It has recently been established that the MBD3-containing NuRD complex is required to modify H3K27me3 by the PRC2 complex within the promoters of the genes participating in development and differentiation processes. Thus, NuRD does not simply repress gene transcription; it is also responsible for the equilibrium between H3K27 acetylation and methylation in embryonic stem cells [102]. However, that is not the only example of interaction between chromatin-remodeling complexes in ESCs. The NuRD complex, namely, its MBD3 subunit, closely interacts with esBAF (Brg1) in mouse ESCs [103]. The Mbd3 and Brg1 subunits colocalize within the transcription start sites and multidirectionally regulate the transcription of a vast gene set. Moreover, Mbd3 and Brg1 play a significant role in transcription regulation through hydroxymethylation of cytosine residues. The MBD3 subunit colocalizes with the TET1 protein and 5-hydroxymethylcytosines (5hmC) *in vivo* ; the binding of MBD3 to promoters is TET1-dependent. *In vitro* experiments have shown that MBD3 binds to 5-hydroxymethylcytosines more efficiently as compared to 5-methylcytosines; knockout of the * Mbd3* gene mostly affects the transcription of 5hmC-marked genes, whereas Mbd3 and Brg1 are required to maintain the 5-hydroxymethylation level [103]. 

5-Hydroxymethylation of DNA was believed to be just an intermediate stage during the demethylation of 5-methylcytosines [104]. However, it turns out that knockout of the genes belonging to the *Tet* family, which encode the proteins performing hydroxylation of 5-methylcytosines, disrupts the differentiation ( *Tet1, Tet2* ) and self-renewal ( *Tet1* ) of ESCs [105–107]. In addition, DNA 5-hydroxymethylation can be retained for a long time during early embryonic development and seems to perform regulatory functions [108, 109]. All these facts indicate that 5-hydroxymethylation can be an independent regulatory state of the epigenome and that NuRD and esBAF play a crucial role in its regulatory potential. Meanwhile, DNA 5-hydroxymethylation directly affects the joint regulatory action of NuRD and esBAF. 

## Tip60-p400 COMPLEX 

The Tip60-p400 complex exhibits histone acetyltransferase and remodeling activity; it can act both as an activator and a repressor of transcription [110, 111]. In addition, Tip60-p400 participates in the replacement of forms of H2AZ-H2B histones [112, 113]. The embryos with a knockout of the *Tip60* and *Trrap* genes that encode the Tip60-p400 subunits die at the pre-implantation stage [114, 115]. The inhibition of the translation of several Tip60-p400 subunits in ESCs via RNA interference has demonstrated that Tip60-p400 is important for the normal self-renewal and differentiation of cells. It has been demonstrated using chromatin immunoprecipitation that p400 colocalizes with NANOG and H3K4me3 (the active chromatin mark) in undifferentiated mouse ESCs. The spectra of the NANOG and Tip60-p400 target genes overlap to a significant extent. Furthermore, NANOG and H3K4me3 are required to provide binding of Tip60-p400 to the target genes. In turn, Tip60-p400 acetylates histone H4 [89]. 

## DIRECT REGULATION OF THE GENES ENCODING THE PROTEINS MODULATING THE CHROMATIN STRUCTURE USING THE TRANSCRIPTION FACTORS PARTICIPATING IN THE MAJOR SYSTEMS OF PLURIPOTENCY MAINTENANCE 

The transcription factors that make up the system of pluripotency maintenance, in addition to their interaction with protein complexes, can directly regulate the genes of chromatin-modifying enzymes. In ESCs, OCT4 activates the demethylase genes Jmjd1a/KDM2A and Jmjd2c/KDM4B, which demethylate H3K9me2 and H3K9me3, respectively, whereas KDM2A and KDM4B, in turn, perform the demethylation of the promoter region of *Tcl1* and *Nanog* , respectively [116]. 

The transcription factors regulating pluripotency interact with the promoters of the genes whose products participate in the global regulation of the chromatin structure. Thus, such factors as OCT4, SOX2, NANOG, SMAD1, ZFX, and E2F1 are associated with the *Chd1 * gene promoter [117]. This gene encodes the enzyme participating in chromatin remodeling. CHD1 binds to histone H3 di- or trimethylated at K4, which is a mark of active chromatin and the genes being transcribed, via two chromodomains [118]. The *Chd1 * repression in mouse ESCs has no effect on the self-renewal of ESCs; however, it tilts the cells towards neural differentiation [119]. 

Factor UTF1 (undifferentiated embryonic cell transcription factor 1), which is transcribed at a high level in undifferentiated mouse ESCs, can participate in the formation of the global chromatin structure. This protein is bound to chromatin; it colocalizes in the regulatory regions of over 1,700 genes, most of which overlap with the previously identified target genes of the transcription factors Nanog, Oct4, Klf4, c-Myc, and Rex1. Reduced synthesis of UTF1 increases the level of expression of most of its target genes and disrupts ESC differentiation. This fact indicates that UTF1 mainly represses the transcription of the genes involved in cell differentiation [120]. It has been demonstrated that the enhancer element localized in the 3’ untranslated region of *Utf1 * binds selectively to Oct4 and SOX2 [121]. 

Thus, regulators of the chromatin structure (CHD1 and UTF1), whose gene expression is directly regulated by the transcription factors that are components of the main internal system of pluripotency maintenance, have been found in ESCs. 

## PLURIPOTENCY AND DNA METHYLATION 

In addition to covalent modifications of histones, DNA methylation is the major mechanism that regulates cellular processes in mammals [122]. Today DNA methylation is known to participate in fundamental phenomena and processes, such as embryogenesis, cell differentiation, genomic imprinting, cancerogenesis, regulation of the transcription of mobile genetic elements, and X chromosome inactivation in female mammals [123–128]. 

DNA methylation indisputably plays a crucial role in the regulation of the self-renewal and pluripotency of cells [129]. Promoters of the major genes associated with the pluripotency maintenance and self-renewal of ESCs ( *Oct4* and *Nanog* ) are hypomethylated in undifferentiated cells and hypermethylated in stem and somatic trophoblast cells [130, 131]. During cell differentiation in a culture or in the embryonic development, promoters of the genes maintaining self-renewal undergo methylation with the participation of the DNA methyltransferases DNMT1, DNMT3A, and DNMT3B [132]. Knockout of the genes encoding the DNA methyltransferases DNMT1, DNMT3A, and DNMT3B causes a disruption of embryonic development and ESC differentiation *in vitro * [132–135]. However, mouse ESCs with simultaneously knocked out genes *Dnmt1* , *Dnmt3a* , and *Dnmt3b* retain their self-renewal ability [136]. DNA methylation performed by DNMT3A and DNMT3B participates in reliable repression of pluripotency genes in embryonic development. Histone methyltransferase G9a, which establishes H3K9me3 within the *Oct4* promoter,recruits the heterochromatin protein HP1 and DNA methyltransferases into this region [137]. 

Cytosine residues in CpG dinucleotides undergo methylation in mammalian genomes [138]. Pluripotent cells are characterized by a reduced methylation level of CpG-rich promoters (containing the so-called GpG islands) and an increased methylation level of CpG-deficient promoters [129, 139]. Most of the CpG-deficient promoters contain H3K4me3, the active chromatin mark. H3K4me3 appears to be established as a result of binding of nonmethylated CpG islands to CPF1 associated with histone methyltransferase SETD1 [140]. In turn, H3K4 methylation can “protect” gene promoters against the impact of DNA methyltransferases [141]. 

It has recently been demonstrated that a significant fraction (up to 25% in human ESCs) of methylated cytosine residues in ESC and iPSC genomes localizes outside CpG [142–144]; non-CpG methylation is predominantly observed in exons rather than in the regulatory gene regions [143, 144]. The pattern of non-CpG methylation in different pluripotent cell lines is very diverse, whereas non-CpG methylation is almost absent in some differentiated cells. Furthermore, knockout of the *DNMT3A* and *DNMT3B * genes in human ESCs drastically reduces the non-CpG methylation level [145]. 

Numerous experimental data indicate that the reprogramming of somatic cells to the pluripotent state (obtaining iPSCs) is accompanied by a global change in methylome towards the state characteristic of pluripotent cells [144, 146, 147]. Promoters of the genes participating in self-renewal maintenance (e.g., *Oct4* and *Nanog* ) undergo demethylation [11, 12, 148]. Such DNA demethylases as TET1 and AID can participate in the reprogramming. Demethylase TET1, which catalyzes the conversion of 5-methylcytosine into 5-hydroxymethylcytosine, is essential for the maintenance of self-renewal of mouse ESCs; it regulates DNA methylation in the *Nanog* promoter [106]. Furthermore, it has been demonstrated using a reprogramming model with mouse embryonic stem/human fibroblast hybrid cells that demethylase AID is required for demethylation of promoters of the human genes *Oct4* and *Nanog * [149]. The fact that the use of inhibitors of DNA methyltransferases allows one to enhance the efficiency of iPSC derivation also lends support to the idea of the significance of methylation for cell reprogramming [146, 150]. 

## PLURIPOTENCY FACTORS IN THE REGULATION OF X CHROMOSOME INACTIVATION 

X chromosome inactivation is a complex process occurring during early mammalian embryogenesis. In mice, imprinted inactivation of the X chromosome inherited from the male parent takes place during the first series of zygote divisions. At the blastocyst stage, the X chromosome is reactivated in ICM cells. Random inactivation of one of the two X chromosomes occurs during gastrulation and differentiation of ICM cells [151–153]. X-inactivation is regulated by a certain locus at the X chromosome, which is known as the X-inactivation center [154]. This locus comprises several genes; however, the *Xist* and *Tsix* genes, which are anti-parallel-transcribed and encode nuclear untranslated RNAs, are considered to be the major regulators [155, 156]. *Xist* RNA was shown to be transcribed monoallelically from the inactive X chromosome, to coat it, and to induce modifications corresponding to inactive chromatin [155]. On the contrary, the *Tsix* gene is a negative regulator of the *Xist * gene; it is transcribed from the active X chromosome [157]. Since X-inactivation takes place during early embryogenesis, an investigation into its dynamics and molecular basis is rather complicated, almost infeasible when humans are used as the objects. Hence, pluripotent cell lines obtained from pre-implantation embryos (ESCs) or by reprogramming mouse or human somatic cells (iPSCs) are currently the most suitable and commonly used models to study X-inactivation. However, studies of the X chromosome status and molecular genetic studies of the regulation of the X-inactivation have revealed a number of differences between mice and humans. 

Embryonic stem cells of female mice derived from pre-implantation blastocysts (3.5 days post coitum) retain a number of the properties of ICM cells; in particular, they can maintain two active X chromosomes in a series of mitotic divisions [152]. Random inactivation of one of the two X chromosomes takes place during the differentiation of mouse ESCs. This property of mouse ESCs is reproducible and stable [152, 153]. 

The situation is more complex for human ESCs, which are also derived from blastocysts (5–9 days post coitum) [4]. A large-scale analysis of a number of human ESC lines has shown that they can be divided into three classes [158]. The first class comprises ESCs with two active X chromosomes, which undergo random inactivation during differentiation; this class corresponds to mouse ESCs. The second class comprises ESC lines in which one of the chromosomes is inactive and the *XIST* gene is transcribed; however, cells retain all of their pluripotency features. The third class contains lines with one X chromosome being inactive; however, the *XIST* gene is not transcribed even after cell differentiation. The inactive X chromosomes in the lines of the second class carry inactive chromatin marks, such as H3K27me3, H4K20me, and the histone variant macroH2A. Interestingly, the lines that belong to the third class carry almost no inactive chromatin marks. Meanwhile, a molecular genetic analysis shows that transcription of most of the genes of the inactive X chromosomes is repressed [158]. 

The fact that pluripotency is not associated with the epigenetic status of X chromosomes in human pluripotent stem cells has also been demonstrated for iPSCs. Mouse iPSCs, similar to ESCs, have two active X chromosomes (in cells derived from females); one of those undergoes random inactivation after the differentiation is induced [159]. However, human iPSCs can have all the features of pluripotent cells and contain an inactive X chromosome; i.e., they can fall into the second class of ESCs [160]. The status of the X chromosome can be changed during reprogramming, resulting in the emergence of subclones corresponding to the first and third classes of ESCs. It has been mentioned that reactivation of the inactive X chromosome can occur during the reprogramming of human somatic cells [147]. In all likelihood, the isolation of clones of ESCs and iPSCs carrying two active X chromosomes can be achieved by varying cell culture conditions. Thus, it has recently been demonstrated that cell culturing under conditions of physiological oxygen concentration (5%) can considerably enhance efficiency in obtaining human ESCs of the first class. On the contrary, transition of the cells to the second and third classes, according to the status of the X chromosome, can be caused by various physiological stress factors [161]. Furthermore, overexpression of *KLF4 * in the presence of a combination of inhibitors of signaling pathways in human ESCs and iPSCs can also cause reactivation of the inactive X chromosome [162]. This fact attests to the instability of the status of the X chromosome in human pluripotent cells. 

**Fig. 4:**
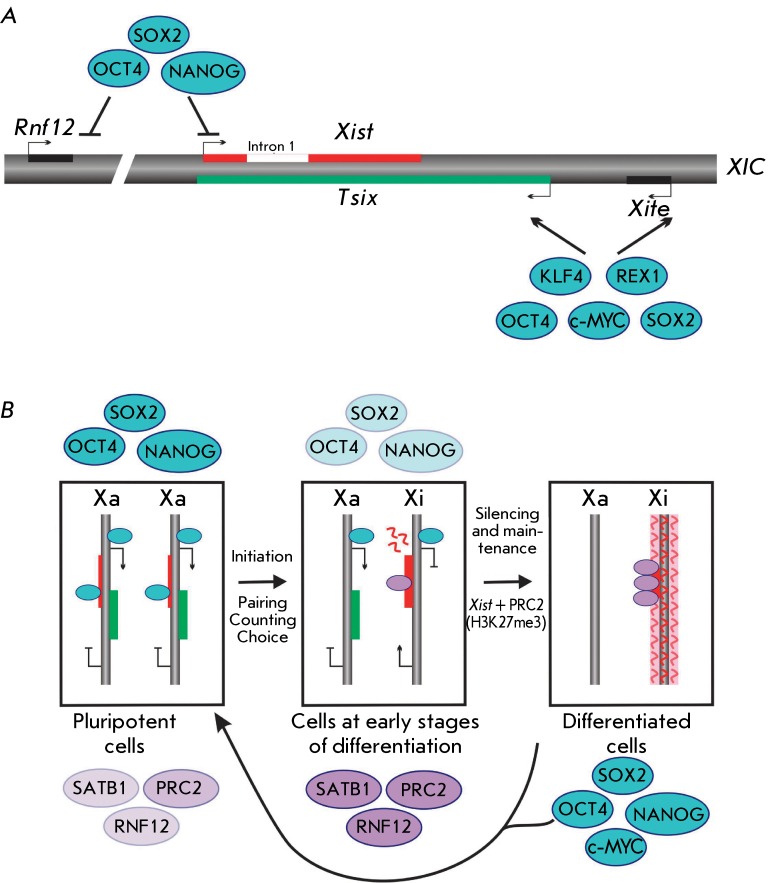
Transcription factors of pluripotency in the regulation of X chromosome inactivation. (A) Scheme of the mouse X chromosome inactivation center (XIC). *Xist* , *Tsix* , and their activators – * Rnf12* and *Xite* – are shown in red, green, and black, respectively. In undifferentiated female mouse ESCs, the transcription factors Oct4, Sox2, and Nanog bind to the first intron of *Xist* and *Rnf12* , repressing their transcription. Meanwhile, OCt4, Sox2, Klf4, Rex1, and с-Myc bind to the regulatory regions of *Tsix* and *Xite* , activating their transcription. (B) In female mouse ESCs, *Tsix* is activated and *Xist* is repressed by the proteins involved in pluripotency maintenance. During the differentiation, one of the X chromosomes is inactivated. X-inactivation is a multistage process including the initiation of inactivation, establishment, and maintenance of transcriptional silencing. Initiation of inactivation occurs due to the decrease in pluripotency factor expression and involvement of chromatin structure regulators (such as SATB1 and PRC2) in the process. Overexpression of Oct4, Sox2, Nanog, and с-Myc in somatic cells induces reprogramming to the pluripotent state, which is accompanied by reactivation of the inactive X chromosome [166]

Despite the fact that the association between the pluripotency of mouse cells and X chromosome status during embryogenesis and in culture is rather obvious, no direct evidence of association between these phenomena at the molecular level had existed until recently. However, the association between transcription factors and regulation of the *Xist* and *Tsix* genes has been revealed. Thus, the transcription factors NANOG, OCT4, and SOX2 have potential binding sites in the first intron of the *Xist * gene and are bound to it in undifferentiated mouse ESCs [163] ( *Fig. 4* ) *.* Knockout of * Oct4* and *Nanog* induces activation of *Xist* transcription. Thus, pluripotency factors can inhibit *Xist * expression via the *Tsix-* independent mechanism [163]. It was established later that the factors NANOG, OCT4, and SOX2 can inhibit *Xist* transcription by repressing the expression of its activator, *Rnf12* . However, the removal of the first intron of *Xist * does not result in X-inactivation [164, 165] ( *Fig. 4* ). 

The factors associated with maintenance of the pluripotency and repression of *Xist * can participate in the activation of *Tsix* transcription [167] ( *Fig. 4* ) *. * Thus, binding of OCT4, SOX2, and KLF4 has been detected within the *Xite* enhancer; although this interaction has not been confirmed in other works [168]. The REX1, c-MYC, and KLF4 binding sites have been detected in the *DXPas34* regulatory element. It has been established that REX1 is required mostly for the elongation of *Tsix* RNA, rather than for the assembly of the transcription complex. Thus, all the aforementioned studies support the fact that the system of pluripotency maintenance is associated with an active status of both X chromosomes in undifferentiated mouse ESCs. Human ESCs do not exhibit these regularities. Human *XIST* transgenes remain active in mouse ESCs despite the presence of pluripotency-maintaining factors. Different mechanisms (e.g., DNA methylation) seem to participate in the regulation of human *XIST* and *TSIX* genes. In mouse ESCs, the *Xist* promoter is only partially methylated even at the active X chromosome; thus, gene transcription is presumably repressed by transcription factors. In human ESCs of the first type, the *XIST* promoter is almost completely methylated (100%). In addition, the differences can be attributed to the fact that the properties of human ESCs (gene expression pattern, sensitivity to signaling molecules) are similar to those of mouse epiblast stem cells, where one X chromosome is inactivated, despite the expression of pluripotency factors [169]. 

In all likelihood, investigations into the status of the X chromosome in human iPSCs should be used in standard tests carried out for newly obtained lines, together with an analysis of the expression of the pluripotency markers that determine the patterns of gene transcription and differentiation. By choosing clones of cells with an inactivated paternal or maternal X chromosome, one can selectively obtain lines with inactive mutant alleles and, hence, cells that can be used to treat X-linked diseases. 

## EPIGENETIC EVENTS OCCURRING DURING CELL REPROGRAMMING TO A PLURIPOTENT STATE. “EPIGENETIC MEMORY” 

Reprogramming of somatic cells to the pluripotent state is accompanied by a global change in their epigenomes [146, 159, 170]. A number of chemical inhibitors of the enzymes participating in the formation of the chromatin structure are currently used to enhance efficiency in generating human and mouse iPSCs. In particular, the use of the histone methyltransferase G9a inhibitor (BIX-01294) and inhibitors of DNA methyltransferases (5’-azacytidine, RG108) and histone deacetylases (valproic acid, TSA, SAHA, sodium butyrate) allows one to increase the reprogramming efficiency tens of times [18, 20, 150, 171–173]. Furthermore, the mechanism of the effect of ascorbic acid (vitamin C) on efficiency in iPSC isolation was recently elucidated [174]. 

**Fig. 5:**
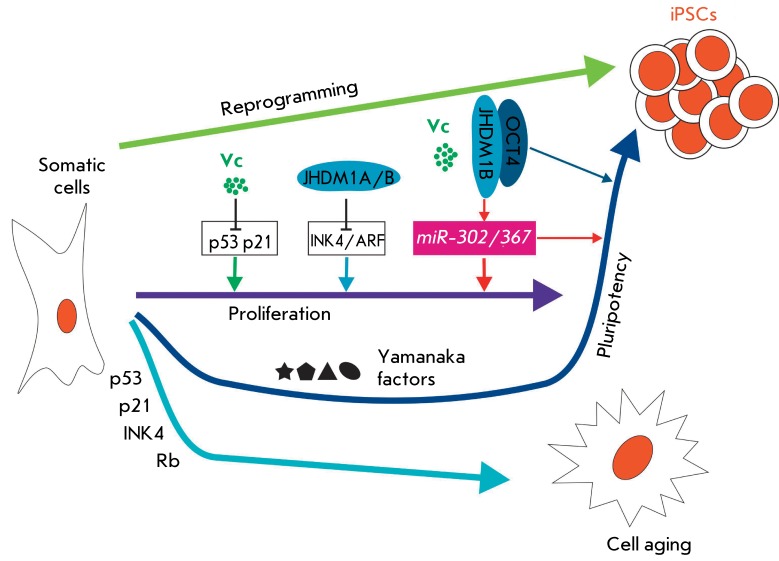
The joint effect of ascorbic acid (vitamin C) and Jhdm1a/1b on the reprogramming of somatic cells to the pluripotent state. Vitamin C and Jhdm1a/1b counteract cell aging by repression of the p53/p21 and Ink4/Arf proteins. In addition, ascorbic acid and the Jhdm1b/Oct4 complex activate expression of the microRNA 302/367 cluster, thus increasing reprogramming efficiency [174]

Ascorbic acid is known to considerably enhance (from 3.8 to 8.75%) the efficiency of reprogramming of fibroblasts and stem cells from adipose tissue; however, its mechanism of action remained unclear [175]. Histone demethylases Jhdm1a and 1b turn out to be the major effectors of ascorbic acid. Ascorbic acid induces Jhdm1a/1b-mediated demethylation of histone H3 at K36 (H3K36me2/3) in a culture of embryonic mouse fibroblasts and during the reprogramming process ( *Fig. 5* ). It has been proven that Jhdm1a/1b are needed for the reprogramming and participate in the acceleration of the cell cycle and inhibition of cell aging via repression of the *Ink4/Arf * locus ( *Fig. 5* ). A high cell division rate and inhibition of the mechanisms of aging and apoptosis are required to provide complete and efficient reprogramming of somatic cells [176–180]. Furthermore, Jhdm1a/1Bs, together with OCT4, activate the expression of the miRNA 302/367 cluster, which is also involved in cell reprogramming [14, 15, 174] ( *Fig. 5* ). 

T. Onder *et al.* [181] have screened a set of interfering RNAs inhibiting the translation of 22 genes whose products participate in DNA and histone methylation. The inhibition of the translation of mRNA of the genes encoding the components of the complexes PRC1 (BMI1, RING1) and PRC2 (EZH2, EED, SUZ12) considerably reduces the efficiency of reprogramming in human fibroblasts. Reduced efficiency was also observed during the inhibition of *EHMT1* and *SETDB1 * encoding H3K9 histone methyltransferases. *YY1* , *SUV39H1* , and *DOT1L * were among the genes in which inhibition of mRNA translation considerably enhanced the reprogramming efficiency. The *YY1* gene encodes a protein acting both as a transcription activator and repressor, depending on the specific context. * SUV39H1 * encodes H3K9 histone methyltransferase; *DOT1L * encodesH3K79 methyltransferase. More attention has been given to *DOT1L* . It turns out that repression of *DOT1L * via RNA interference or chemical inhibition of DOT1L can substitute the functions of *KLF4* and *c-MYC* in experiments for generating iPSCs from human fibroblasts. In addition, inhibition of DOT1L at the early stages of reprogramming results in the activation of *NANOG* and *LIN28* , which are also used in the case of human cells. A genome-wide analysis the H3K79me2 distribution has demonstrated that the genes associated with epithelial-mesenchymal transition, whose expression is specific to fibroblasts, lose this histone modification at the early stages of reprogramming. DOT1L inhibition accelerates deletion of H3K79me2 within the genes subjected to transcriptional silencing in iPSCs [181]. 

All these facts attest to a crucial role played by the system of epigenetic regulators in the reprogramming process. 

High-performance analysis methods were used to reveal a high degree of similarity between iPSCs and ESCs in terms of the gene expression pattern and epigenomic state both at the level of DNA methylation and distribution the covalent histone modifications H3K27me3 and H3K4me3 [147, 182]. 

Despite significant similarity between iPSCs and ESCs at the molecular level, it has been demonstrated in a series of studies that the transcriptomes and epigenomes of individual iPSC lines can possess certain common features and retain a number of characteristics that are intrinsic to the original somatic cells [183–186]. The phenomenon of retaining certain features of the epigenomes of somatic precursors is known as epigenetic memory [187, 188]. 

The modern methods of molecular genetic analysis allow one to carry out high-resolution genome-wide studies of DNA methylation and distribution of covalent histone modification. The study by R. Lister *et al* . [144], which employed the Methyl-C-Seq technique, can be given as an example. This technique allows one to carry out genome-wide studies of cytosine methylation at single-nucleotide resolution. The authors have tried to avoid the possible effect of the method of obtaining iPSCs and types of somatic cells on the results obtained. Five iPSC lines were used in this study: one line was obtained via retroviral transduction of adipose tissue cells with *OCT4, SOX2, KLF4* , and *c-MYC* ; the second line was obtained via lentiviral transduction of lung fibroblasts IMR90 with *OCT4, SOX2, NANOG* , and *LIN28* ; and three lines were obtained from foreskin fibroblasts using non-integrating episomal vectors. Furthermore, two ESC lines and trophoblast derivatives of iPSCs and ESCs differentiated using BMP4 were included in the study. The methylation status of 75.7–94.5% of all cytosine residues in the genomes of 11 cell lines has been determined. It is of interest to note that the authors have focused on not only methylation of cytosines within CpG dinucleotides but on non-CpG-methylation as well (CpH, where H = A, C or T). It has been shown that at the genomic scale, human iPSCs and ESCs have similar methylation patterns. The genomes of pluripotent cells tend to be more methylated (on average) than those of somatic cells. Serious differences at the level of CpH-methylation of DNA have been revealed. Somatic cells, including adipose tissue stem cells, are characterized by an extremely low level of such type of methylation; whereas the share of methylated cytosines within CpH dinucleotides in DNA in iPSCs and ESCs is 20–30% of the total amount of methylated cytosine residues in the genome. Moreover, enrichment of exons and introns in methylated CpH is observed both in ESCs and iPSCs. 

It is interesting to mention that despite the general similarities between the methylomes of ESCs and iPSCs, a number of differences between them have been revealed, including 1,175 differentially methylated regions (DMRs) with a length varying from 1 to 11 thousand base pairs (the total length being 1.68 million bp). No DMRs have been detected between two ESC lines analyzed under the same conditions. Differentially methylated regions in ESCs and iPSCs can be subdivided into two groups. The first group contains DMRs whose emergence can be attributed to inheritance of the methylation pattern of the somatic precursor cells of iPSCs (44–49% of the total number of DMR). The second group contains DMRs whose methylation pattern is specific to iPSCs (i.e., differs from the DMR pattern both in somatic cells and in ESCs). DMRs of this kind make up 51–56% of the total number of detected DMRs. DMR distribution varies in five of the iPSC lines that have been analyzed: 62% occur in two lines out of five; 16% occur in all five lines. These regions can be regarded as “hotspots” of epigenetic reprogramming, which require increased attention when obtaining iPSCs. A significant number of DMRs (80%) are associated with CpG islands; 62% localize near the genes or in the genes; 29 and 19% lie within 2 thousand bp from the transcription start or termination sites, respectively. A bioinformatic analysis of the function of the genes localized near DMRs and occurring in all the iPSCs under analysis showed no marked predominance of the genes involved in certain cellular processes. This attests to the fact that methylation disturbance during the reprogramming can affect a large number of cellular functions. Another important regularity is the predominance of hypomethylation in DMRs (109 out of 130, 92%) in all five lines. The disturbances in methylome reprogramming when obtaining iPSCs can be attributed to insufficient methylation. 

DMRs have also been detected by an attentive analysis and comparison of CpH methylation in ESCs and iPSCs. A total of 29 regions have been found; they are characterized by the extensive length (half of these regions is over 1 million bp long; the longest one is 4.8 million bp); the total length of CpH-DMR is 32.4 million bp. Most CpH-DMRs in iPSCs are hypomethylated as compared with ESCs; they localize near centromeres and telomeres. These regions are enriched in histone H3 trimethylated at K9 (H3K9me3) and colocalized with hypermethylated CpG-DMRs. Most genes localized in these regions are characterized by an increased level of methylation of promoter regions and, therefore, by a reduced transcription level. It is interesting that the level of the inactive chromatin mark (H3K27me3) is reduced in these regions. Thus, extensive domains associated with the near-centromeric and near-telomeric regions with aberrant distribution of histone modifications, disturbed patterns of CpG and CpH methylation, and a disturbed level of gene transcription, have been revealed in human iPSCs. These “hotspots” of epigenomes undoubtedly need to be subjected to a thorough investigation when obtaining new human iPSC lines [144]. 

An investigation into CpG methylation in 22 human iPSC lines derived from various somatic cells (endometrial cells, umbilical vein epithelial cells, amnion cells, fetal lung fibroblasts, and menstrual blood cells) has also revealed differences from ESCs [186]. 1,459 differentially methylated CpG sites corresponding to 1,260 genes were detected when comparing all iPSC and ESC lines using a DNA microchip containing probes for 24,273 CpG sites within 13,728 genes. However, the number and distribution of these sites in different iPSC lines varied considerably. The reason may be that the lines were obtained from somatic cells of different types. In more than 15 lines out of 22, only 20 sites were shared. It is worth noting that the number of these sites was increased in XX iPSCs. The comparison of these data with the results obtained by R. Lister *et al* . [144] has revealed 72 differentially methylated promoters in both studies. However, according to [186], most DMRs in iPSCs were hypermethylated as compared to ESCs; hence, it was postulated that the iPSC genome is methylated to a higher extent. On the contrary, R. Lister *et al.* [144] have reported hypomethylation of CpG dinucleotides in iPSCs. However, these differences can be attributed to the features of the experimental approaches used. In particular, K. Nishino [186] has analyzed the CpG localized mostly within the CpG islands in the promoter regions of the genes, whereas R. Lister *et al* . determined cytosine methylation in the entire genome. Furthermore, it has been clearly demonstrated [186] that the level of aberrant hypermethylation at later passages (30–40) is considerably lower than that at earlier ones (4–6), whereas R. Lister *et al* . [144] used iPSC lines which had undergone tens of passages. 

The surprising similarity between the transcriptomes and epigenomes of these cells and those of ESCs was emphasized in the early studies devoted to obtaining mouse and human iPSCs. Furthermore, it has been demonstrated that the gene transcription pattern in somatic cells at the genome-wide level changes to the maximum extent. However, it was established later that iPSCs retain certain (often rather insignificant) features of somatic transcriptomes and epigenomes [187, 188]. Despite its apparent unimportance, incomplete reprogramming of particular loci can considerably affect the properties of pluripotent cells by changing their differentiation ability. Thus, a significant similarity between mouse ESCs and iPSCs at the level of mRNA and miRNA transcription (with the exception of several transcripts) has been detected [189]. In particular, aberrant silencing of the imprinted *Dlk1-Dio3* locus has been observed in certain iPSC clones, including those derived from hematopoietic precursor cells, which are also characterized by a low transcription level of this locus. This effect is assumed to be caused by the “epigenetic memory.” Due to the transcriptional disturbance in the *Dlk1-Dio3 * locus iPSCs become incapable of efficient formation of chimera and cannot form the mouse organism via tetraploid complementation. It is interesting to mention that treatment with valproic acid, the histone deacetylase inhibitor, leads to transcription activation in the *Dlk1-Dio3* locus and restores the iPSC capability of tetraploid complementation and efficient formation of chimeric animals [189]. 

A number of interesting studies have been devoted to the investigation of the effect of the origin of iPSCs on their differentiation pattern [185, 186, 189, 190]. Thus, the properties of iPSCs derived from mouse hematopoietic, neuronal precursors and fibroblasts were compared with those of ESCs. Embryonic stem cells originated either from blastocysts obtained by nuclear transfer from somatic cells or from those obtained by natural fertilization. First, it turned out that the type of somatic cells strongly affects efficiency and quality in reprogramming. The molecular genetic parameters of iPSCs derived from hematopoietic cells were much closer to those of ESCs, whereas fibroblast-derived iPSCs gave rise only to partially reprogrammed clones. iPSCs derived from neuronal precursors were the closest to ESCs. Second, the differences between iPSCs and embryo-derived pluripotent cells have been revealed via the analysis of DNA methylation. Similar to the earlier studies, it has been established that iPSCs and embryo-derived pluripotent cells differed by a large number of DMRs. iPSCs obtained from neural precursors and fibroblasts are characterized by residual methylation of the loci responsible for the formation of the hematopoietic line, which causes a decreased differentiation level of these iPSCs in the corresponding direction. Third, the limitations on the directions of differentiation of iPSCs of a certain origin can be eliminated. If iPSCs derived from neuronal precursors are differentiated into hematopoietic cell lines and secondary iPSCs are subsequently obtained from these derivatives, these secondary iPSCs will have a higher potential towards differentiation into blood cells. Furthermore, the impact of the inhibitors of histone deacetylases and DNA methylation (such as trichostatin A and 5-azacytidine) on the epigenome can considerably reduce the effect of the cell origin on their differentiation [187]. It should be mentioned that iPSCs at very early passages were used in [187]. Aberrant cytosine methylation at early passages and, therefore, disruption of the pattern of gene expression and iPSC differentiation have also been revealed in other studies. Thus, it has been demonstrated that mouse iPSCs derived from fibroblasts, B lymphocytes, bone marrow granulocytes, and precursor cells of skeletal muscles possess “epigenetic memory,” which is manifested at the transcriptional level and results in differentiation predominantly into the cell types from which they had been obtained [190]. It has been established that the genes that are markers of certain somatic cells can continue being expressed at a high level in pluripotent cells, with the inactive chromatin marks (H3K27me3) in their promoter regions being reduced and active chromatin marks (H3Ac and H3K4me3) being increased. No differences in the methylation of the promoters of these genes have been observed [190]. It is significant that these transcription disturbances and shifts in cell differentiation are eliminated after long-term cultivation of the iPSCs clones. These data, along with the results of other studies, attest to the fact that reprogramming is a gradual process; the establishment of the completely reprogrammed state of epigenome and cells in general requires a large number of rounds of genome replication. 

In addition to the studies focused on the disturbance of epigenome reprogramming and “epigenetic memory” in mouse iPSCs, several papers have already been published, which have confirmed the fact that a similar phenomenon exists in the reprogramming of human cells. It has been demonstrated that iPSCs derived from neuronal precursors and β cells of the pancreatic gland and human retinal pigment epithelium can have a non-random differentiation pattern; i.e., the direction of differentiation is strongly tilted towards the precursor type of somatic cells [188, 191, 192]. Aberrantly methylated regions have also been detected in iPSCs derived from umbilical cord blood cells and neonatal keratinocytes, and the existence of the “epigenetic memory” has been established, which consists in predominant differentiation into parent-type cells and is retained even after a large number of passages [193]. 

Thus, the problem of “epigenetic memory” today remains among the major hurdles in the derivation and application of induced pluripotent stem cells. It is a pressing problem, especially due to the fact that iPSCs display great potential for use in regenerative medicine and as models for human diseases. Resolution of this problem will not only enable efficient usage of human and animal iPSCs for biomedical purposes, but can also provide new fundamental knowledge on the organization and role of cell epigenomes in culture and during the embryonic development of organisms. 

## Abbreviations

ESC: embryonic stem cells
iPSC: induced pluripotent stem cells
DMR: differentially methylated regions
ICM: inner cell mass
